# Notes on Dental Hygiene

**Published:** 1887-06

**Authors:** George Robinson

**Affiliations:** Oarnam, Otago, New Zealand


### Notes on Dental Hygiene.—Chiefly Addressed to Young Practitioners.

BY GEORGE ROBINSON, OARNAM, OTAGO, NEW ZEALAND.

That dental hygiene in the surgery and laboratory and their various contingencies are still imperfectly attended to, I have for many years been convinced, and will feel obliged if you will allow me a little space in your valuable JOURNAL to air some of my views upon this subject, when convenient to you, and subject to your scrutiny.

It is very surprising, to me a matter of query, how those dentists manage to afford any time for cleanliness who charge such ridiculously small fees for extracting teeth, and for other operations; their general surroundings must surely be very imperfectly attended to, and must sooner or later become, to put it mildly, rather unwholesome, and conducive to the diseases of both their own teeth and impairment of their health in various ways, perhaps undreamed of by the majority. The most scrupulous cleanliness in connection with our work, instruments, and general surroundings is of the utmost importance to both dentists and their clients.

Instruments should be thoroughly cleansed by the intelligent use of suitable antiseptics, as 10 per cent. of carbolic acid, and tincture of iodine, when that is indicated, and liquor potasii permanganatis ; such precautions are specially needed when the instruments have been used for persons in bad health, or when certain diathes are even slightly suspected. To some persons precautions may appear unnecessary, but to those who are by microscopical research acquainted with bacteria, it must be otherwise. A case of ozœna may pass unobserved by many, while to others it is so unmistakably proclaimed, that it is quite oppressive. After operating in such cases, great relief is afforded to the offended olfactory sense by the liberal use of a little Condy's fluid in water in which the operator performs his evening ablutions after work. This every wise operator will not fail to do most thoroughly. For some antiseptic purposes menthol is, I believe, superior to all other agents.

The use of disinfectants or antiseptics should not be relied upon in any case as a substitute for thorough cleanliness. They are poor, indeed, unless supplemented by ample supplies of fresh air in the laboratory and surgery, and general hygiene. The flame of a spirit lamp, cautiously applied to an instrument which has been wiped after using, may prevent possible contagion.

A denture which has been worn, and is presented for repair, addition, or other purpose, very often needs a decided antiseptic treatment before being manipulated in the least. Fresh, dry garden earth is very good. Solution of chloralum is useful to immerse plates in; but sometimes more potent means are desirable. As an instance I may mention a recent case which was presented to my notice. The most objectionable one I have seen for many years, since ivory went out of use, was a set on celluloid, made eighteen months ago, by a respectable practitioner, and with most recent improvements in the pivot; the plate of the lower set, particularly, was completely perforated along the buccal and lingual surfaces, as if perforated by a "Teredo Dentalis," being porous on close inspection, with the unaided sight. What would a microscopic exploration reveal? Myriads of bacteria, living, dying, etc., etc. This is one of several cases which I have seen which seem to confirm all the statements of the people who are practically acquainted with the unreliable character of celluloid. Messrs. Ash & Son, of London, than whom no more trustworthy authority exists, on dental materials, have informed me, some twelve months ago, that it has been abandoned both in Great Britain, the Continent of Europe, the United States, India, &c. No doubt the (very erroneous) supposed advantages which cheap dentistry affords a deluded public, have brought this and other perishable bases into use; its beauty is but skin deep, and it fades accordingly, and becomes wrinkled early, and probably also do those who wear it, and directly or indirectly suffer more or less impaired health, both from the unsanitary condition of the mouth and also from the disappointment and worry consequent upon imperfect mastication. No amount of scrubbing could remove the unhealthy secretions with which such cases become loaded. A large number of persons are unable to detect such conditions, at least for a long time, owing to their defective sight, until aided by powerful magnifiers, which reveal the hidden mysteries of the oral inhabitants, and compel the most sceptical to admit the truth of Hamlet's well-known exclamation. There can be no doubt that the greater care is necessary to ensure perfect sanitation in everything connected with the art and science of dentistry, and to satisfy the demands of an exacting public, as well as for the comfort of the practioner's own conscience, and thus by avoiding these causes of disease and worry, the dentist's own life would be greatly prolonged.

Young dentists of a few years' practice evidently do not always see the necessity of such precautions until they have felt the effects of unsanitary conditions; but neglect will with the busy pursuit of our calling tell its own tale, whether it is perceived or not.

On reference to the Odontological Society's Journal of Transactions, I observe Mr. Oakley Coles has dwelt exhaustively on the subject of maintenance of health in dental practitioners, and many of those excellent remarks I can confirm from personal experience and observation, and I may add I have found great benefit from the use of a Lyon's stool, which for some twelve years or so I have had in use, otherwise I would have been unable to continue my work before I came to this colony, where I have had a great deal more leisure than work, and being much stronger, have little need to use it.

By way of introduction to the concluding portion of my notes on dental hygiene, allow me to say that it is with the idea and belief that dental practitioners are advancing, and not retrograding, that I venture to write. Were it not so I might remain silent Some of my remarks may not coincide with popular ideas and current opinions, yet I am led to hope that careful and unprejudiced investigation will gain some adherents to the tenets which I hold. The evidence, on which I believe my statements and propositions rest, are constructed from varied materials, abstract and concrete, as they arise in my mind and appear to support my views. And, as I take it to be an admitted fact that the immense benefits which have been hitherto derived from improved sanitation are amply established, those and other reasons must plead my excuse if I appear somewhat enthusiastic in urging the claims of sanitary reforms, and greater attention to some minor details—more especially in connection with the practice of dental art and science Any one who has read the life of Louis Pasteur will be more prepared, perhaps, to admit the necessity of a consideration of those malarious influences which are no longer mysterious, save to the most ignorant, and are now known to cause many diseases which proper cleanliness will prevent; the neglect of *minor ailments* often lead to *major ones.* Many are, no doubt, too busy to give much attention to hygiene, but having observed for a number of years men drop out of the ranks just as they become experts or soon after, and, in many instances, a little more healthful recreation in the pure open air daily would have prolonged valuable lives many years. Slaving away at an arduous profession will not fail to produce physical infirmities in some form by the invasions of unobserved causes stealthily causing irreparable mischief, which only the strongest constitutions can resist for any great length of time.

According to some writers in the United States of America, the life of a dentist who is busy at the chair is quite brief; yet, bad as overwork may be, is not worry worse? Hygiene should, I think, receive more attention than it does. One of the most important, yet perhaps most neglected, aids to the analysis and detection of sanitary surroundings is the high cultivation of the olfactory sense, which can be improved to a degree of acuteness far beyond that which is commonly possessed. Among the best means that can be employed to secure this faculty I believe to be attention to general hygiene, the use of *pure aseptic* food, more attention to, and observance of the various odors peculiar to chemical substances, gases, compounds, etc., etc., including fermentation products.

The use of pungent narcotics, as tobacco; strong perfumes, as musk, *patchouli*, and similar perverting odors, when much used are antagonistic to the attainment of a high degree of olfactory sensibility.

The microscope is a most valuable aid which can not well be dispensed with in the discovery of malific germs, not that all germs are generators of disease, for some are undoubtedly consistent with perfect health; but the septicity which results from neglect of some of the laws of sanitation can not fail to be ultimately productive of disease in some form, and to curtail the legitimate length of life.

It is my firm conviction that very *few persons* live nearly *as happily, healthfully,* or long as they might do. True, we are told by the poet—

To desire not to live long, but well,
How long we live, not years, but actions tell.

So far, so good; yet to live healthy lives is more consistent with happiness, and calculated to prolong our existence and extend our usefulness.

It appears to me that every thing which is calculated to produce a gross or impure condition of body is antagonistic to true enjoyment of life and health; therefore, general hygiene, as treated of in the works of Drs. Wilson and Ed. A. Parkes, etc., should, I think, receive some attention by every dental practitioner. One of the most important branches of hygiene is the thorough ventilation, not only of all surgeries and laboratories, but of every part of *every dwelling;* a want of which will negative all efforts in the direction of health and purity. A Tobin's ventilator, etc., is very excellent, I believe, for carrying off such contaminated air as floats near it, *when* there is a *proper intake* near the basement to admit fresh air which is to cause a current. This is, I have generally observed, by opening a door or window; but in order to *thoroughly flush* a room it is necessary to provide some special contrivance which shall act constantly, yet can be shut off when required, and so arranged as to take in air from as pure a source as possible, and on the *sunny side* of the house in *cold weather,* or the opposite in warm weather, when required. This is obviously more practicable in some countries than in others. The difficulty of controlling currents of cold air—which, in Great Britain, at least, are not desirable—may, I believe, be met by attention to the foregoing points, and the admission of air through some intercepting medium, which will prevent too much draught in cold or windy weather. Such a medium I have devised. A machine could easily be constructed to produce the desired perforations, which hitherto I have only been able to produce imperfectly, yet have had a strip of zinc so perforated in constant use in my surgery, and also a similar one in my laboratory, with decided advantage, for two years. As I can have the upper part of the window open, to the extent of six or eight inches, without feeling the draught on my head—the unpleasantness of which leads to my contrivance, which differs from ordinary perforated zinc by the perforations being in a slanting direction, and the little flaps causing the air to be deflected in any direction you require, in the most simple and inexpensive manner. In warm weather when I wish to admit more air, I have only to pull the sash down lower, etc.

While the complete in-breathing of *pure air* is of the utmost importance to man, yet it is very rarely accomplished; and in the best dwellings even of the highest nobility, ventilation is generally imperfect, ALMOST universally so. To the student of practical hygiene it is a matter of surprise that so little improvement has been effected in this direction. To the mass of the people all sanitary progress is exceedingly slow. It is equally important, both for the health of the practitioner, as well as his client's comfort, that his clothing and all his surroundings be scrupulously clean. No doubt, in the majority of cases this is attended to; but there are many, I fear, where there is ample room for improvement. Finally, are there not physical reasons why some people do not perceive, or are unconscious of, the existence of malaria? How many fatal cases we have read of, where people have been crowded more or less; yet, had they been aware of their danger, would they not have avoided it? These instances indicate that we may breathe poison and yet not be conscious of it—nay, utterly oblivious, when accustomed to small doses; for it is well known our bodies are very accommodating, and health is easily *simulated* until a certain boundary line is passed, which line varies with the peculiarities of the individual's constitution, and some perhaps slight ailment precedes more serious disease, which is not always traceable to the true cause. And these remarks apply to mental as well as physical hygiene. I think we should aim at the highest mental and physical health which is attainable. The old state of indifference, which very strong people may regard with apparent impunity, unsanitary conditions will not be tolerated much longer.

In order to secure anything like good health every person requires a certain amount of daily exercise in the open air; but if we are too much fatigued by overwork, we can not enjoy life as every honest man is justly entitled to. It is perfectly absurd for any man to be a mere working machine, a drudge, whose chief relaxation, in many instances, consists in drawing poisonous fumes into his mouth, and then puffing them out again immediately, and perhaps flattering himself that he has soothed his jaded nerves by means of a harmless agent, which may not be quite so innocent as it seems, and its benefits very questionable indeed, and is not consistent with what I take to be hygienic conditions when employed daily to the extent that is customary. And equally inconsistent with true hygiene is the daily use of alcoholic stimulants, which are perhaps quite as deceptive as foul air and tobacco; though to a jaded man there is a strong temptation to take a little of *something*, you know. And indeed I do not condemn a man, but pity the conditions which cause him to require such flimsy crutches.

For various reasons I must now conclude my present notes, which, if they do no present good, may possibly help on a little towards the end in view, viz., *mens sana in corpore sano.—British Journal Dental Science.*

